# Improving the Viability and Metabolism of Intestinal Probiotic Bacteria Using Fibre Obtained from Vegetable By-Products

**DOI:** 10.3390/foods10092113

**Published:** 2021-09-07

**Authors:** María Ángeles Rivas, María José Benito, Santiago Ruíz-Moyano, Alberto Martín, María de Guía Córdoba, Almudena V. Merchán, Rocío Casquete

**Affiliations:** 1 Nutrición y Bromatología, Escuela de Ingenierías Agrarias, Universidad de Extremadura, Avd. Adolfo Suárez s/n, 06007 Badajoz, Spain; mrivasm@unex.es (M.Á.R.); srmsh@unex.es (S.R.-M.); amartin@unex.es (A.M.); mdeguia@unex.es (M.d.G.C.); avmerchan@unex.es (A.V.M.); rociocp@unex.es (R.C.); 2 Instituto Universitario de Investigación en Recursos Agrarios (INURA), Avd. de la Investigación, Universidad de Extremadura, 06006 Badajoz, Spain

**Keywords:** dietary fibre, by-product valorisation, probiotics, prebiotic

## Abstract

This study evaluated the effect of dietary fibre obtained from pomegranate, tomato, grape and broccoli by-products on the gastrointestinal transit survival, growth, and metabolism of six probiotic strains. The results showed that the studied by-products contained variable amounts of polysaccharides that affected the six probiotic microorganisms in different ways. In addition, the protective effect of the fibre obtained on the probiotic strains was more effective in the case of the fibre obtained from tomato peel. In terms of growth, grape stems showed the best results, favouring the growth of lactic acid bacteria. Finally, all fibres were able to increase the content of short-chain fatty acids in the in vitro test, but broccoli stems and pomegranate peel stimulated higher production of short-chain fatty acids. The results of this study demonstrate that plant by-product fibres can improve survival, growth, and metabolism in terms of the fatty acid profiles of probiotic strains, highlighting the desirability of harnessing these by-product fibres to develop new high-value-added ingredients as probiotic carriers.

## 1. Introduction

Plant-based food production generates large amounts of food waste during processing and storage, which are referred to as by-products that could be reutilized to avoid environmental risks and economic losses [1]. These residues comprise vegetable waste and by-products such as skins, stems, and seeds. Vegetable by-products are rich sources of dietary fibre (DF) and an important source of soluble polysaccharides. Polysaccharides from agro-food industry residues constitute one of the most important renewable resources. The wide variety in their chemical composition and structure, as well as their biodegradability and safety, make them suitable for applications in many fields such as food, pharmaceuticals, cosmetics, tissue engineering, biofuels, and others [2,3]. These are, therefore, bioactive compounds that are gaining increasing interest in their use as food additives or supplements, focusing efforts on improving their biological activity by new extraction methodologies [4].

Polysaccharides from agricultural residues are part of plant cell walls, which are highly diverse in their structure and composition [5]. In general, cell walls are constituted by high-molecular-weight polysaccharides, which are mainly lignin, cellulose, hemicelluloses, pectins, and other non-starch polysaccharides such as inulin and oligosaccharides [6]. Cell-wall-forming polysaccharides are classified into insoluble and soluble polysaccharides based on their solubility in water. Insoluble polysaccharides are lignin, cellulose, hemicelluloses, and pectins (insoluble); the soluble polysaccharide group consists of other pectins, and hemicelluloses [7].

Pectins are mostly considered as soluble fibre and are a polygalacturonic acid-rich part of the plant cell wall that can be composed of up to 17 different monosaccharides [8]. They are also the most structurally complex natural plant polysaccharides [9], and numerous health benefits are attributed to them [10]. They can be obtained from many vegetable sources, primarily citrus peel and apple pomace, but are also present in tomato peel [11,12], pomegranate peel [13], grape skin [14], and broccoli stems [15].

One of the most important activities of DF is its prebiotic activity, which can selectively stimulate the activity or growth of probiotic microorganisms in the colon [16]. In this regard, research efforts have been directed towards the inclusion of these prebiotics in daily dietary foods, most notably in dairy products [17], but also in beverages, processed fruits and vegetables, bakery, confectionery, snacks, sweeteners, and baby formula [18]. Due to this growth-stimulating activity of probiotic microorganisms, the addition of prebiotics and probiotics together to foods is more attractive because it promotes the viability and stability of the microorganisms [19]. Probiotics, aided by prebiotics, have a beneficial effect on consumer health by improving microbial balance at the gut level [20]. To provide these benefits, probiotics must remain viable, with a total number of live organisms above 10^6^ cfu/g of food. The survival of these microorganisms during food processing and storage as well as during intestinal transit is clearly compromised on many occasions [21]. Several technologies have been employed to improve the viability of these microorganisms exposed to environmental stress and intestinal transit, such as encapsulation, in which different methodologies have been used depending on the drying temperature: high temperature drying (spray drying and fluid bed drying) and low temperature drying (ultrasonic vacuum spray drying, spray cooling, electrospinning, supercritical technique, freeze drying, extrusion, emulsion, enzymatic gelation, and impact spray technique) [22].

An alternative method is to combine probiotic cells with fibrous materials where probiotic cells can be stuck to or even form biofilms on the surface. This would confer high viability after gastrointestinal digestion [23,24]. Only a few studies are available in the literature on the use of fibre-rich agronomic by-products as protectors of probiotics as well as prebiotics for growth and metabolism stimulation [25].

The aim of this work was to characterize the fibre obtained from tomato, pomegranate, grape, and broccoli by-products and to investigate the effects of this fibre on the intestinal transit survival, growth, and metabolism of six strains with probiotic properties in order to develop new prebiotic or symbiotic food ingredients.

## 2. Materials and Methods

### 2.1. Plant Material

In this study, four agro-industrial by-products were used, including pomegranate peel, tomato peel, grape stalk, and broccoli stem. The vegetable by-products used in this work were provided by horticultural plants from the Extremadura region of Spain. All samples were dried in a forced-air oven at 45 °C for 24–48 h, ground, packed in vacuum bags, and stored at room temperature until use.

### 2.2. Sample Preparation

DF concentrates were prepared using the alcohol-insoluble residue method described by Femenia et al. [26], with modifications. Briefly, three replicates (5 g each) of dry samples were homogenized with 85% (*v/v*) ethanol. The mixture was boiled on a shaker for 10 min, and then the solid residue was collected using a Büchner funnel with cellulose-free filters (Whatman, 934-AH ™ glass microfiber filters, Sigma Chemical Co., St Louis, MO, USA). This process was repeated twice, the final time with absolute ethanol. Finally, the insoluble solid residue was washed with acetone, and the excess solvent was removed after 24 h at room temperature.

### 2.3. Determination of Neutral Sugar and Uronic Acid

To determine the content of uronic acids and the profile of neutral sugars, the DF was previously solubilized (autoclave treatment at 121 °C for 16 min followed by an ultrasound treatment for 1 h), and the soluble content of the DF extract was precipitated by adding to the supernatant 3 times its volume of absolute ethanol at 60 °C. Then, it was centrifuged, and the supernatant was removed. The solid residue was dried in an oven at 45 °C. Once dry, the soluble residue of the DF extract was subjected to a hydrolysis process with 12 M sulfuric acid (3 h at room temperature and 100 °C for 1 h). Finally, the monosaccharides and galacturonic acids released were determined by HPLC. HPLC analyses in this study were conducted using a 1260 Infinity II LC Agilent HPLC System (Waters, Milford, MA, USA), which consisted of a separation module, RI detector. The HPLC system was equipped with a Rezex-ROA column (7.8 mm internal diameter × 150 mm; Phenomenex, Torrance, CA, USA). In isocratic mode, the mobile phase was water, at a flow rate of 0.6 mL/min. In elution mode, the sample injection volume was 10 μL, the column temperature was 80 °C and the detector temperature was 40 °C.

### 2.4. Survival of Probiotics in the Presence of DF Extracts

Co-incubation of probiotics and DF extracts was performed in MRS broth supplemented with 1% DF extracts. The bacterial strains used were *Lactobacillus casei* (HL 245, HL 233) (Spanish type culture collection), *Lactobacillus reuteri* (PL503, PL519), *Enterococcus faecium* (SE 906, SE 920) [27,28]. Then, 10 mg of DF was weighed out and mixed with 900 µL of (de Man, Rogosa and Sharpe) MRS broth, which was sterilised at 121 °C for 15 min, cooled, and inoculated with 100 μL of MRS broth sterile with probiotic inoculum content of 10% (10^9^ cfu/mL) in aseptic conditions. Co-incubation was carried out over 18 h at 37 °C at 180 rpm with orbital shaking to ensure a homogeneous distribution of the DF extract supply. As a blank, probiotics incubated without DF extracts were used.

A standardised in vitro static digestion method was performed for simulated gastrointestinal digestion [29]. Simulated gastric juice was mixed with an equal volume of probiotic suspensions. A 1 M HCl solution was used to adjust the pH of these samples to pH 2 prior to gastric digestion at 37 °C over 2 h. Following gastric digestion, the gastric mixtures were then mixed with an equal volume of simulated intestinal juice and adjusted to pH 7 with a solution of 1 M NaOH prior to intestinal digestion at 37 °C for 6 h. When gastric and intestinal digestion were completed, viable bacteria were counted in MRS medium in the digested samples after 0, 2 and 6 h. The data were expressed in logarithmic reduction of cfu/mL with respect to the initial inoculum (Time 0 h: 8 Log cfu/mL).

### 2.5. In Vitro Prebiotic Capacity

The prebiotic capacity of the soluble DF extracts was assessed using the method described previously by Ruiz-Moyano et al. [30]. Inoculum preparations consisted of aliquots of stock cultures of the bacteria strains *L. casei* (HL 245, HL 233) (Spanish type culture collection), *L. reuteri* (PL 503, PL 519), and *E. faecium* (SE 906, SE 920) (Ruiz-Moyano et al. [27,28] grown in Man–Rogosa–Sharpe (MRS; Scharlab, Barcelona, Spain) broth over 24 h at 37 °C. The strains were tested for growth in the presence of solubilized DF (autoclave treatment at 121 °C for 16 min followed by an ultrasound treatment for 1 h). Five microlitres of each bacterial suspension strain was inoculated into 200 μL of semi-solid MRS medium which contained 0.125 g/L agar, glucose-free and supplemented with 2 g/L of each sterile-filtered extract as the exclusive carbohydrate source. Semi-solid MRS supplemented with 2 g/L glucose was used as a positive control for growth, and other DF control was made with short-chain fructo-oligosaccharide (FOS), whereas the negative control was a carbohydrate-free semisolid MRS. Turbidity was measured in a fluorimeter (FLUOstar OPTIMA F), growth was carried out for 48 h at a temperature of 37 °C and readings were taken at a wavelength of 570 nm at 1-h intervals. The ability of each strain to grow was evaluated by comparing, in percentage, the growth in each extract with that in the control.

### 2.6. Determination of Short-Chain Fatty Acids Produced in the Presence of Fibre Extracts

To determine the ability to produce short-chain fatty acids (SCFAs), lactic acid bacteria (LAB) strains that were grown with the improved fibre extracts were selected. They were grown in modified MRS broth at 37 °C. The MRS was prepared as a commercial MRS without glucose and sodium acetate and complemented with 2 g/L of carbohydrate source (improved fibre extracts). Culture supernatants were obtained by centrifugation of the medium at 8000× *g* for 5 min before filtration through 0.22-µm filters (Thermo Fisher Scientific, Waltham, MA, USA).

To measure the amount of SCFAs, 500 µL of supernatant was mixed with 500 µL of ultrapure water and 100 µL of internal standard (2-ethylbutyric acid). Then 0.5 µL was injected into a gas chromatograph with a split/split-less injector and a flame ionisation detector (Shimadzu 2010 Plus). SCFAs were separated on a DB-FFAP capillary column (30 m × 0.25 mm id; 0.25 µm). The initial oven temperature was maintained at 80 °C for 2 min, and then increased to 200 °C at 20 °C/min and maintained for 12 min. The injector and detector were set at 250 °C. Helium at 1.8 mL/min was the carrier gas. Individual SCFAs were determined by comparing their retention times with those of the reference standard mixtures from Sigma (Sigma Chemical Co., St Louis, MO, USA). SCFA concentrations were determined as the ratio of the peak area of the analyte to the internal standard (2-ethylbutyric acid), according to Brighenti [31].

### 2.7. Statistical Analysis

Statistical analysis of the data was carried out using SPSS for Windows, version 21.0 (IBM Corp., Armonk, NY, USA). Descriptive statistics of the data were determined, and the differences within and between groups were studied by one-way analysis of variance (ANOVA) and separated by Tukey’s honestly significant difference test (*p* ≤ 0.05). Principal component analysis (PCA) was performed on the correlation matrix of the variables. We worked with three biological replicates and each of them were analysed in triplicate.

## 3. Results and Discussion

### 3.1. Fibre Constituents: Neutral Sugars and Pectins

The results of the DF composition of by-products of tomato and pomegranate peels, and grape and broccoli stems are shown in Table 1.

The most abundant components of the DF extracts were uronic acids (which indicate the pectin content), and their values ranged from 581.24 to 934.72 mg of galacturonic acid per gram of fibre extract. Tomato peel exhibited the highest values, 934.72 mg/g, showing significant differences with the rest of the by-product extracts studied. Regarding the neutral sugar profile, the main monosaccharides were glucose and fucose in tomato and pomegranate peel, mannose in the case of grape stems, showing significant differences with the rest of the by-products, and arabinose and xylose in broccoli stems. In general, it was observed that galactose and rhamnose were the minority sugars in all analysed samples.

The results obtained for pectin concentration were higher than those found by other authors. Depending on the extraction method, Sengar et al. [32] obtained between 675.8 and 913.3 g/kg of galacturonic acid in tomato peel. Pomegranate peel was described with a lower amount of pectins, between 377 and 755 mg/g uronic acids [33]. Lower amounts of pectins were also found in grape and broccoli stems, ranging from 31.09 mg/g in grape stems [14] to 159.5 mg/g in broccoli stems [34].

The profile of the majority of neutral sugars was variable depending on the by-product. Glucose levels in tomato and pomegranate skins were significantly higher than those in grape and broccoli stems. This glucose may come from non-pectic polysaccharides or be a remnant of soluble sugars that were not completely removed during the extraction procedure, and other sugars identified in other studies coincide, although in different amounts, with those found in our study [14,32,33,34].

### 3.2. Survival of Probiotics in the Presence of DF Extracts

Table 2 shows the results obtained from the assay performed to check the ability of the DF extracts to protect the probiotic strains used from gastrointestinal tract transit. The data are expressed as Log cfu/mL reduction with respect to the initial inoculum (Time 0 h: 8 Log cfu/mL).

In the case of *E. faecium* SE 920, it was observed that tolerance to low pH and gastrointestinal transit was superior in the presence of the different DF extracts, and the best behaviour was observed in the presence of tomato peel extract, although it improved in all cases with respect to the control without fibre. In the same way, tomato peel extracts were able to improve survival through the gastrointestinal tract in all strains of *L. casei* and *L. reuteri*. In addition, the survival of all strains was improved more or less in the presence of DF, except that *E. faecium* SE 906 did not tolerate the low pH and gastrointestinal transit in vitro in the absence of extract, in the control or in the presence of the different DF extracts. Tomato DF extract presented the highest pectin concentration, which may be related to its protective activity. Anal and Singh [35] studied several biopolymers generally recognized as safe as encapsulation materials, including gelatine, pectin, and alginate, for their ability to improve the viability and shelf life of probiotic bacteria. Other authors have shown that multi-layered emulsions including pectins extracted from citrus peels can be used to incorporate probiotics into various products and improve their viability during processing, storage, and after ingestion [36]. Blaiotta et al. [37] demonstrated that chestnut fibre was able to greatly improve the tolerance of *Lactobacillus* to simulated gastric and bile juice.

Soluble DF can create a viscous environment for probiotic bacteria, providing a protective effect and enhancing cell adhesion or biofilm formation in the fibre matrix [38].

### 3.3. In Vitro Prebiotic Capacity

Table 3 shows the capacity of the six probiotic bacterial strains tested for in vitro growth on four DF extracts. The results showed that the DF extracts from the grape stem sample achieved the highest growth values for all the strains studied, *E. faecium* SE 920 showing the highest growth (107.29%). In addition, significant differences were found in the growth of the *E. faecium* SE 906, *E. faecium* SE 920, *L. casei* HL 245, and *L. casei* HL 233 strains in the presence of the grape stem fibre extracts with respect to the rest of the extracts. However, no significant differences were found in the growth of the two *L. reuteri* strains. On the one hand, pomegranate peel extracts achieved moderate growth of *L. casei* HL 233 and *E. faecium* strains (48.35% and 44.49%, respectively). On the other hand, all the strains studied showed a slight growth in the presence of tomato peel and broccoli stem extracts.

Polysaccharides such as pectin are formed by groups of complex structure, which are metabolised more slowly. Therefore, the ability of bacteria to metabolize fibres of this subgroup as an energy source will be influenced by the degree of polymerization, molecular weight, chain size, and the presence of branching in the molecule [39]. The speed and rate of prebiotic DF fermentation by the gut microbiota depends on several factors such as solubility, chain size, porosity, total particle surface area, and the structure and organization of the fibre cell wall. Furthermore, the fermentation outcome of a fibre mixture is not the same as that of each individual DF [40]. In addition, other dietary components such as proteins, lipids, and phenolic compounds can affect the fermentability and prebiotic effect of DF [41].

### 3.4. Short-Chain Fatty Acids Produced in the Presence of Fibre Extract Production

Table 4 shows the production of short-chain fatty acids of the probiotic bacteria grown in the presence of the different DF extracts studied. No significant differences were found in the production of the different fatty acids at the strain level. However, significant differences were found according to the type of extract used. Acetic acid was the major acid in all cases, and the values ranged from 14.39 to 355.59 mM. The highest production of acetic acid was observed in the presence of broccoli stem extract, which showed significant differences with respect to the other extracts, followed by grape stem and pomegranate peel samples. By contrast, tomato skin showed the lowest value for acetic acid production. Overall, it can be stated that pomegranate skin achieved the highest values for the other minority fatty acids, followed by the broccoli stem and grape stem samples; however, as in the case of acetic acid, the tomato skin DF extracts showed the lowest values. The glucose and FOS controls obtained average values for all the fatty acids studied.

Baenas et al. [42] observed that the production of fatty acids in the presence of DF extracted from raspberry was generally increased during fermentation. Similar to the results obtained by us, previous studies revealed that DF extracted from potato residue could promote the production of acetic acid, butyric acid, isobutyric acid, valeric acid, and isovaleric acid [43].

### 3.5. Multivariate Analysis of the Parameters Related to DF Extract Studied from Different Subproducts

**Figure 1:**
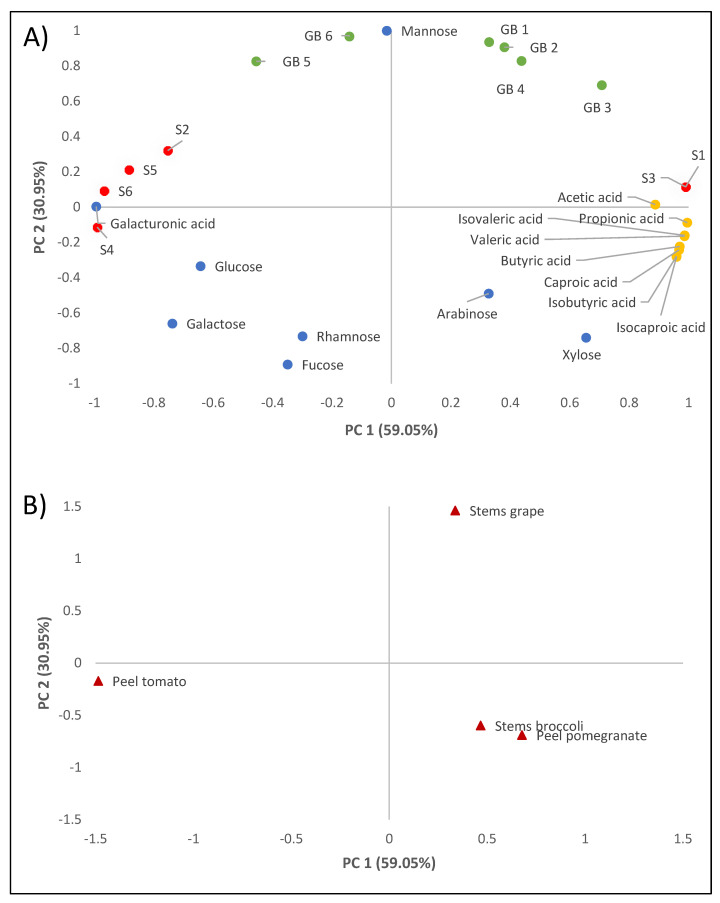
Principal component analysis of the analytical results of the dietary fibre extracts studied. Loading plot (**A**). GB1: growth of *E. faecium* SE 906; GB2: growth of *E. faecium* SE 920; GB3: growth of *L. casei* HL 245; GB4: growth of *L. casei* HL 233; GB5: growth of *L. reuteri* PL 503; GB6: growth of *L. reuteri* PL 519; S1: survival in the gastrointestinal tract of *E. faecium* SE 906; S2: survival in the gastrointestinal tract of *E. faecium* SE 920; S3: survival in the gastrointestinal tract of *L. casei* HL 245; S4: survival in the gastrointestinal tract of *L. casei* HL 233; S5: survival in the gastrointestinal tract of *L. reuteri* PL 503; S6: survival in the gastrointestinal tract of *L. reuteri* PL 519. Score plot (**B**). Grape stem, broccoli stem, pomegranate peel, and tomato peel samples.

PCA was carried out for the entire set of DF data to obtain an interpretable overview of the main information. Figure 1 shows the two-way loadings and score plots, where PC2 was plotted against PC1, explaining 90% of the total variance. According to Figure 1A, PC1 best explains the difference between the variables of bacterial survival to gastrointestinal transit and the production of short-chain fatty acids such as propionic acid, while PC2 shows the variability between the neutral sugars present in the soluble fibre extract.

With regard to the factors studied, principal component analysis clearly shows how the fibre extracts obtained from the different by-products behave differently with respect to the variables studied. The fibre extracted from grape stems was positively correlated with the percentage of lactic acid bacterial growth with respect to the positive control, while the fibre extracted from broccoli stems and pomegranate peels was correlated with the production of short-chain fatty acids. On the other hand, tolerance to low pH and gastrointestinal transit was superior when tomato peel extract was present.

## 4. Conclusions

This study presents a characterization of the effect of DF obtained from pomegranate, tomato, grape, and broccoli by-products on the gastrointestinal transit survival, growth, and metabolism of six probiotic strains. These by-products were shown to have high concentrations of polysaccharides that affected the probiotic microorganisms studied in different ways. The protective effect on intestinal transit of the fibre obtained from tomato skin was superior to that of the other extracts of pomegranate, grape, and broccoli. The fibre extracted from grape stems favoured the growth of lactic acid bacteria, and the fibre extracted from broccoli stems and pomegranate peel stimulated the increased production of short-chain fatty acids. The results reveal that these by-products can be considered as high-quality sources of DF for joint applications with probiotic microorganisms by aiding and stimulating their survival, growth, and metabolism, and their use as prebiotics in the food industry would help to develop new high value-added ingredients.

## Figures and Tables

**Table 1:** Neutral sugar and uronic acid profile of dietary fibre from vegetable by-products (mg/g dry weight).

Parameters	Tomato Peel			Pomegranate Peel			Grape Stems			Broccoli Stems		
	Mean		SD ^1^	Mean		SD	Mean		SD	Mean		SD
Neutral sugar (mg/g*)*												
Glucose	6.04	±	1.37 ^b^	4.7	±	1.00 ^b^	1.35	±	0.01 ^a^	0.56	±	0.00 ^a^
Rhamnose	0.26	±	0.00 ^c^	0.12	±	0.04 ^b^	<0.1 ^a^			0.34	±	0.00 ^d^
Xylose	<0.1 *^a^			2.17	±	0.46 ^b^	<0.1 ^a^			2.37	±	0.02 ^b^
Mannose	0.97	±	0.31 ^a^	0.48	±	0.12 ^a^	2.33	±	0.01 ^b^	0.62	±	0.00 ^a^
Fucose	3.09	±	0.70 ^b^	3.09	±	0.03 ^b^	1.65	±	0.01 ^a^	2.61	±	0.18 ^ab^
Galactose	1.54	±	0.53 ^b^	0.84	±	0.22 ^ab^	<0.1 ^a^			0.69	±	0.00 ^ab^
Arabinose	1.33	±	0.00 ^a^	1.47	±	0.15 ^a^	1.20	±	0.01 ^a^	3.40	±	0.02 ^b^
Uronic acids (pectin) (mg/g*)*												
Galacturonic acid	934.72	±	45.81 ^b^	581.24	±	53.91 ^a^	655	±	0.32 ^a^	657.51	±	4.58 ^a^

^1^ SD, standard deviation; * The limit of detection was 0.1 mg/g; ^a,b,c^ Values with different superscripts are significantly different (*p* ≤ 0.05) between samples.

**Table 2:** Tolerance of bacterial strains to low pH, and complete gastrointestinal transit. Data are expressed in Log cfu/mL reduction with respect to the initial inoculum (Time 0 h: 8 Log cfu/mL).

Strains	Extracts	pH Tolerance (2.5)	Tolerance to Gastointestinal Transit
		2 h	6 h
*E. faecium* SE 906	Control	8	8
	Tomato peel	8	8
	Pomegranate peel	8	8
	Broccoli stems	8	8
	Grape stems	8	8
*E. faecium* SE 920	Control	3	8
	Tomato peel	0	1
	Pomegranate peel	1	4
	Broccoli stems	1	2
	Grape stems	2	2
*L. casei* HL 245	Control	2	5
	Tomato peel	2	4
	Pomegranate peel	2	2
	Broccoli stems	2	2
	Grape stems	2	2
*L. casei* HL 233	Control	2	2
	Tomato peel	0	1
	Pomegranate peel	2	2
	Broccoli stems	1	2
	Grape stems	2	2
*L. reuteri* PL 503	Control	1	2
	Tomato peel	0	0
	Pomegranate peel	1	2
	Broccoli stems	1	1
	Grape stems	0	1
*L. reuteri* PL 519	Control	3	5
	Tomato peel	2	2
	Pomegranate peel	3	5
	Broccoli stems	4	4
	Grape stems	3	4

**Table 3:** Percentage growth of lactic acid bacteria (LAB) strains, with respect to the positive control, on soluble dietary fibre extracts.

	*E. faecium* SE 906			*E. faecium* SE 920			*L. casei* HL 245			*L. casei* HL 233			*L. reuteri* PL 503			*L. reuteri* PL 519		
Extracts	Mean		SD ^1^	Mean		SD	Mean		SD	Mean		SD	Mean		SD	Mean		SD
Control (FOS ^2^)	7.21	±	0.53 ^a^	27.97	±	0.65 ^a^	68.14	±	0.78 ^c^	28.75	±	0.77 ^a^	29.05	±	0.06 ^ab^	7.60	±	10.4 ^a^
Tomato peel	8.88	±	0.19 ^a^	23.29	±	4.35 ^a^	14.16	±	6.44 ^a^	23.95	±	3.07 ^a^	31.21	±	0.50 ^ab^	25.38	±	1.28 ^ab^
Pomegranate peel	10.34	±	0.96 ^a^	44.49	±	0.45 ^b^	38.23	±	1.25 ^b^	48.35	±	1.66 ^b^	21.52	±	1.55 ^a^	16.06	±	0.46 ^ab^
Broccoli stems	15.53	±	0.82 ^b^	26.84	±	0.03 ^a^	30.36	±	1.94 ^b^	24.09	±	6.95 ^a^	26.95	±	6.55 ^ab^	21.81	±	0.22 ^ab^
Grape stems	42.31	±	2.71 ^c^	107.29	±	1.32 ^c^	58.12	±	4.58 ^c^	82.90	±	2.26 ^c^	34.27	±	0.45 ^b^	37.58	±	9.94 ^b^

^1^ SD, standard deviation; ^2^ FOS, fructo-oligosaccharides; ^a,b,c^ Values with different superscripts are significantly different (*p* ≤ 0.05) between samples; Negative growth (≤20%); Slight growth (>20%–≤40%); Moderate growth (>40%–≤70%); High growth (>70%).

**Table 4:** Production of short-chain fatty acids in mM of lactic acid bacteria (LAB) in the presence of different soluble fibre extracts.

	Acetic Acid			Propionic Acid			Isovaleric Acid			Butyric Acid			Isocaproic Acid			Isobutyric Acid			Valeric Acid			Caproic Acid		
	Mean		SD ^1^	Mean		SD	Mean		SD	Mean		SD	Mean		SD	Mean		SD	Mean		SD	Mean		SD
*Extracts (E)*																								
Control (Glucose)	70.90	±	57.00 ^b^	0.51	±	0.34 ^abc^	0.37	±	0.21 ^bc^	0.06	±	0.03 ^ab^	0.69	±	1.46	0.08	±	0.06 ^ab^	0.40	±	0.21 ^bc^	0.24	±	0.17 ^ab^
Control (FOS ^2^)	85.15	±	26.30 ^b^	0.41	±	0.07 ^ab^	0.35	±	0.11 ^abc^	0.04	±	0.01 ^a^	0.18	±	0.07	0.06	±	0.01 ^ab^	0.30	±	0.13 ^b^	0.17	±	0.06 ^ab^
Tomato peel	14.39	±	10.70 ^a^	0.08	±	0.03 ^a^	0.11	±	0.03 ^a^	0.01	±	0.00 ^a^	0.00	±	0.00	0.02	±	0.00 ^a^	0.00	±	0.00 ^a^	0.03	±	0.04 ^a^
Pomegranate peel	221.40	±	27.27 ^c^	1.13	±	0.45 ^d^	0.83	±	0.30 ^d^	0.13	±	0.05 ^c^	0.72	±	0.37	0.22	±	0.12 ^d^	0.91	±	0.29 ^d^	0.61	±	0.28 ^c^
Broccoli stems	355.59	±	150.39 ^d^	1.06	±	0.47 ^cd^	0.76	±	0.31 ^d^	0.13	±	0.00 ^c^	0.64	±	0.35	0.19	±	0.09 ^c^	0.82	±	0.39 ^d^	0.59	±	0.30 ^c^
Grape stems	256.37	±	52.94 ^c^	0.89	±	0.22 ^bcd^	0.61	±	0.12 ^cd^	0.09	±	0.02 ^bc^	0.42	±	0.15	0.13	±	0.03 ^bc^	0.63	±	0.17 ^cd^	0.41	±	0.12 ^bc^
*Strains (S)*																								
*E. faecium* SE 906	161.49	±	110.94	0.61	±	0.49	0.51	±	0.33	0.07	±	0.05	0.36	±	0.35	0.12	±	0.12	0.47	±	0.37	0.33	±	0.27
*E. faecium* SE 920	153.71	±	116.08	0.63	±	0.49	0.45	±	0.31	0.07	±	0.05	702	±	1.36	0.11	±	0.09	0.47	±	0.37	0.32	±	0.27
*L. casei* HL 245	184.06	±	212.62	0.67	±	0.58	0.49	±	0.39	0.08	±	0.07	0.37	±	0.39	0.11	±	0.11	0.53	±	0.46	0.34	±	0.34
*L. casei* HL 233	163.86	±	137.21	0.62	±	0.42	0.46	±	0.28	0.07	±	0.05	0.34	±	0.31	0.10	±	0.08	0.45	±	0.36	0.31	±	0.27
*L. reuteri* PL 503	136.30	±	106.83	0.72	±	0.63	0.43	±	0.29	0.06	±	0.05	0.31	±	0.29	0.09	±	0.08	0.44	±	0.34	0.27	±	0.25
*L. reuteri* PL 519	149.20	±	89.38	0.81	±	0.54	0.46	±	0.30	0.07	±	0.05	0.34	±	0.30	0.11	±	0.08	0.96	±	0.37	0.31	±	0.26
*Values P*																								
*Pe*	0.000			0.000			0.000			0.000			0.031			0.000			0.000			0.000		
*Ps*	0.230			0.850			0.952			0.957			0.576			0.960			0.952			0.983		
Pe*s	0.000			0.929			0.876			0.960			0.796			0.995			0.982			1.000		

^1^ SD, standard deviation; ^2^ FOS, fructo-oligosaccharides; ^a,b,c,d^ Values with different superscripts are significantly different (*p* ≤ 0.05) between type of extracts or strains. *Pe*: *p*-value of the extracts; *Ps*: *p*-value of the strains; Pe*s: *p*-value of the interaction between extracts and strains.

## Data Availability

Not applicable.
